# Expression of human lambda expands the repertoire of OmniChickens

**DOI:** 10.1371/journal.pone.0228164

**Published:** 2020-01-29

**Authors:** Kathryn H. Ching, Kimberley Berg, Jacqueline Morales, Darlene Pedersen, William D. Harriman, Yasmina N. Abdiche, Philip A. Leighton

**Affiliations:** 1 Ligand Pharmaceuticals Incorporated, Emeryville, California, United States of America; 2 Carterra, Inc., Salt Lake City, Utah, United States of America; Monash University, AUSTRALIA

## Abstract

Most of the approved monoclonal antibodies used in the clinic were initially discovered in mice. However, many targets of therapeutic interest are highly conserved proteins that do not elicit a robust immune response in mice. There is a need for non-mammalian antibody discovery platforms which would allow researchers to access epitopes that are not recognized in mammalian hosts. Recently, we introduced the OmniChicken^®^, a transgenic animal carrying human VH3-23 and VK3-15 at its immunoglobulin loci. Here, we describe a new version of the OmniChicken which carries VH3-23 and either VL1-44 or VL3-19 at its heavy and light chain loci, respectively. The Vλ-expressing birds showed normal B and T populations in the periphery. A panel of monoclonal antibodies demonstrated comparable epitope coverage of a model antigen compared to both wild-type and Vκ-expressing OmniChickens. Kinetic analysis identified binders in the picomolar range. The Vλ-expressing bird increases the antibody diversity available in the OmniChicken platform, further enabling discovery of therapeutic leads.

## Introduction

Since the first monoclonal antibody (mAb) therapies were approved over 30 years ago, the antibody therapeutics space has continued to expand to an increasing number of indications in oncology, autoimmunity and infectious disease [1]. According to the most recent report by the Antibody Society, 864 unique antibody-based therapies, either in development or already approved, addressed 884 different clinical indications, demonstrating the vast landscape targeted by antibody technologies [2]. As the antibody therapeutics space expands, tools to develop and identify potential antibody candidates have grown increasingly sophisticated. From early efforts to engineer chimeric antibodies or introduce humanizing mutations, to transgenic animals carrying human V, D and J genes, the platforms and engineering tools available to generate therapeutic candidates with greater human content continue to evolve.

Hybridoma technology enabled researchers to retain the heavy and light chain pairings that had undergone repeated rounds of somatic hypermutation and selection *in vivo*, and the development of transgenic animals reduced subsequent *ex vivo* engineering required to make the resulting antibodies more human-like and therefore less immunogenic in the clinic [3–5]. Transgenic animal platforms available to generate these antibodies have traditionally been mammalian platforms. When an antigen is highly conserved between mammalian species, however, discovery requires alternative strategies. Because of their evolutionary and phylogenetic distance from mammals, avian species, and in particular, chickens, offer an alternative strategy when self-tolerance to the immunogen is a concern [6,7]. Historically, the use of chicken as an antibody discovery platform has been limited both by the lack of a fusion partner to immortalize chicken B cells and the technical knowledge to introduce human transgenes into the chicken genome. We circumvented the lack of a chicken fusion partner by employing a microdroplet technology to isolate antigen-specific B cells and perform single cell RT-PCR [8]. Further, we recently described the OmniChicken^®^, the first transgenic chicken with fully human V genes at its immunoglobulin loci [9]. The first generation OmniChicken carried pre-rearranged VK3-15*01+JK4 and either a pre-rearranged or a rearranging VH3-23*01+D1+JH4 or JH6 at its light and heavy chain loci, respectively. Unlike most mammals, V(D)J rearrangement is not the primary mechanism of generating diversity in the chicken. Rather, diversity derives from the process of gene conversion which utilizes an array of pseudo VH (ψVH) and pseudo VL (ψVL) genes upstream of the functional V [10,11]. ψVH and ψVL genes are promotor-less and lack recombination signal sequences. During the gene conversion process, stretches of homologous sequence in the ψVs replace sequence in the rearranged V. Interestingly, because this type of diversification requires stretches of homology, hypervariability is concentrated in the CDRs and not framework regions [12]. The pseudogenes of the original OmniChicken were derived from somatic human sequences in the Expressed Sequence Tags (EST) database. The constant regions were all chicken. We demonstrated that the OmniChicken could generate antibodies to brain-derived neurotrophic factor (BDNF), which is 97% conserved between humans and mice, and 91.5% conserved between humans and chickens. In addition to producing antibodies against a conserved mammalian target, the OmniChicken displayed similar epitope coverage of a model antigen compared to wild-type chickens and mice [13]. A significant number of clones were also mouse cross-reactive even though only human protein was used as immunogen.

The decision to use a VK3-15 gene for the development of OmniChickens was based on its expression in HEK293 cells and its frequency in naturally occurring human repertoires [9,14]. Vκ light chains also represent 70% of light chains in clinically approved antibody therapeutics [15], which likely stems from the bias toward kappa light chains in the wild-type mouse, where many of the antibodies were originally discovered and subsequently humanized. The native light chain locus of the chicken, however, consists of a single Vλ gene which most closely resembles the mammalian Vλ [10]. In the OmniChicken, expression of a chimeric light chain consisting of human Vκ and chicken CL did not limit the antibody repertoire of the animal. However, these birds did have slightly lower total plasma IgY compared to wild-type chickens [9]. Intrachain (i.e. V_H_/CH1 and V_L_/CL) and interchain (i.e. V_H_/V_L_ and CH1/CL) interactions have been studied extensively in the case of fully human or fully mouse antibodies [16,17]. In an elegant study using a variety of Fab and scFv assemblies, Röthlisberger et al. evaluated the contributions of each domain to the stability of the molecule [18]. Using fluorescence spectroscopy to measure protein unfolding, they found stabilization occurred primarily through interchain interactions. While the constant regions do contribute to the overall stability, they do so mainly as a unit, not through intrachain contacts. In the case of the OmniChicken, we have paired human V-regions with chicken constant domains. To investigate to what extent the chimeric nature of the OmniChicken IgY affected plasma IgY levels, and whether Vλ would produce levels closer to wild-type since human Vλ is more similar to the native chicken light chain, we generated two Vλ insertion vectors: SynVL-D, based on VL3-19, inserted upstream of chCL, and SynVL-E, based on VL1-44, inserted upstream of huIGLC3 and therefore producing a fully human light chain. We also assessed the antibody repertoire and immunocompetence of these new transgenic lines.

## Results

### Insertion of lambda transgene

Two SynVL insertion vectors were generated. The SynVL-D insertion vector contained a functional VL3-19*01 + JL2*01 and chicken constant region. Human VL3-19 shares the most homology with the chicken lambda gene compared to all other light chain human Vs (67% at the amino acid level). The SynVL-E insertion vector contained a functional human VL1-44*01 + JL3*01 upstream of human IGLC3*01 (S1 Fig). VL1-44 is one of the most frequently expressed Vλ genes in the naturally occurring human repertoire [14]. To generate diversity, an array of human pseudogenes based on a VL3-19 or a VL1-44 framework was designed for SynVL-D and SynVL-E, respectively. CDR diversity was derived from naturally occurring, somatic human ESTs found in the Express Sequence Tags database (NCBI). Diversity was concentrated in the CDRs with minimal changes in the framework regions of the pseudogenes (S2 Fig). Like our previously described Vκ insertion, all noncoding regions of the transgenes were chicken, including enhancer elements and promotor sequences. These Vλ-expressing chickens were then bred to birds with either a pre-rearranged human VH3-23 (SynVH-C) or rearranging VH3-23 (SynVH-SD) at the chicken heavy chain locus, both previously described [19], and combined with knockouts on the other allele for each locus, producing birds with the genotype SynVL/IgLKO; SynVH/IgHKO.

### Vλ OmniChickens show normal lymphocyte populations in the periphery

**Fig 1:**
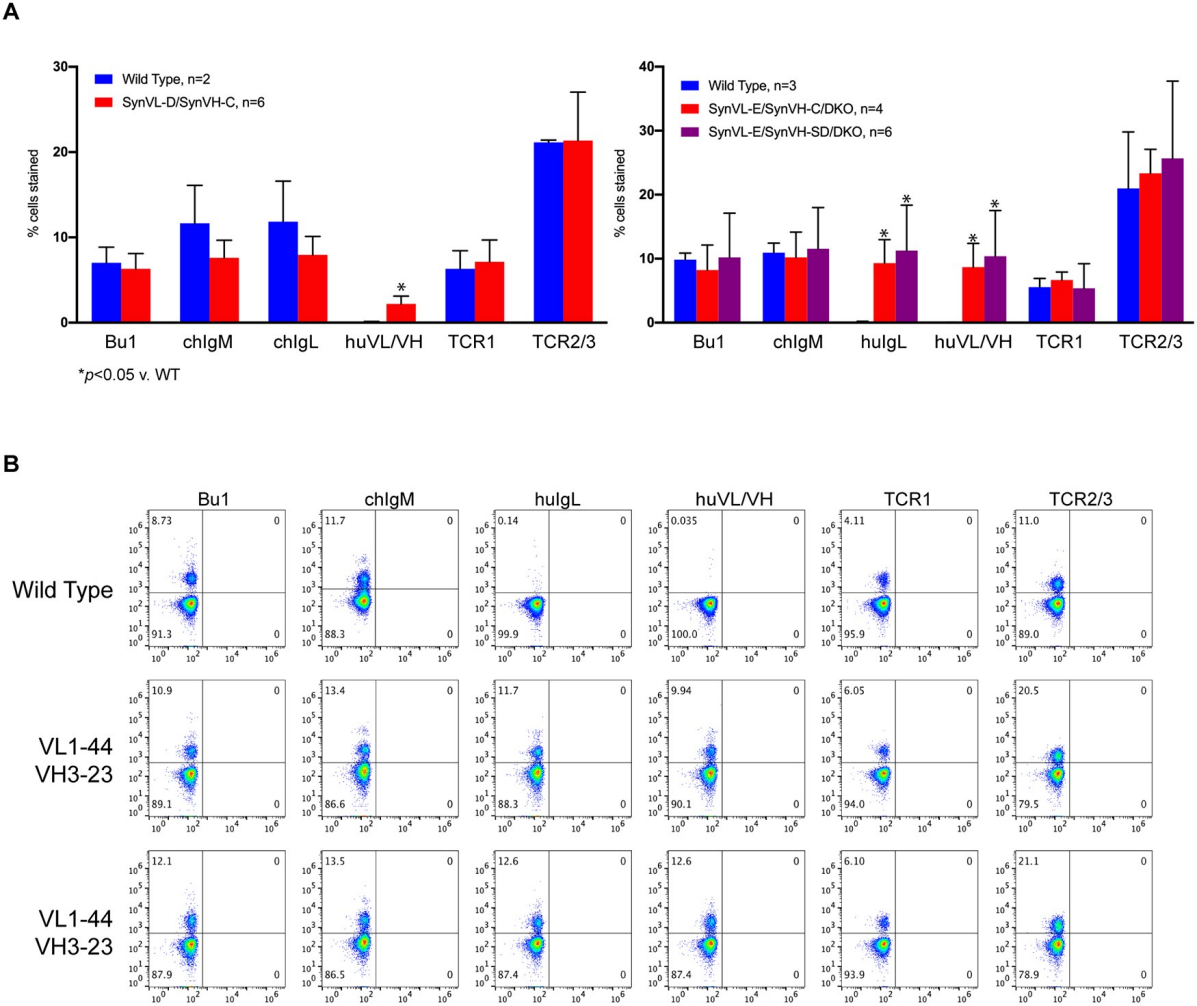
Vλ-expressing OmniChickens demonstrate normal B cell populations in the periphery. (A) Circulating lymphocytes were isolated from SynVL-D (left panel) and SynVL-E OmniChickens (right panel) and stained for Bu1, a chicken B cell marker, chIgM (μ-specific), chIgL (chCL specific), huIgL (huCL specific) and two different T cell markers. Additionally, a polyclonal antibody raised against the inserted human transgenes was used (huVL/VH). Populations were compared to age-matched wild-type chickens, and no difference in B and T cell populations were observed (Student’s T-test, p>0.05). Error bars represent the standard deviation from the mean. (B) Representative plots from SynVL-E OmniChickens show a distinct population of cells expressing surface Ig, and two human specific antibodies demonstrate expression of the human V at the cell surface. Data are representative of several independent experiments.

At five weeks of age, lymphocytes from wild-type, SynVL-D OmniChickens with SynVH-C and SynVL-E OmniChickens with either SynVH-C or SynVH-SD at the heavy chain locus were isolated and labeled for Bu1 (chicken B cell marker), chicken IgM (chFc specific), chicken IgL (chCL specific), human IgL (huCL specific), a human V-region specific antibody and two different T cell markers, TCR1 and TCR2/3, then analyzed by flow cytometry. Compared to wild-type birds, lambda OmniChickens, regardless of which human heavy chain was present, expressed comparable numbers of lymphocytes in the periphery (Fig 1A). A Student’s T-Test demonstrated no significant difference between any of the Vλ birds and wild-type birds for Bu1, chIgM, TCR1 and TCR2 (Table 1). SynVL-D expressing birds stained strongly with the constant region-specific anti-chicken IgL antibody (Fig 1A, left panel, compared to WT, *p* = 0.21), as would be expected as it expresses chCL. Because the constant region of the SynVL-E insertion is human, no staining with the anti-chicken IgL antibody was observed (S3 Fig) but staining with an anti-human CL antibody was very robust (Fig 1A, right panel and Fig 1B). We also raised rabbit polyclonal antisera against the human VH3-23, which is expressed by both SynVL-E and SynVL-D birds, and VL1-44 insertions (in a single chain format with human Fc). Both lambda OmniChickens stained with this antibody (huVL/VH), but not the wild-type birds (Fig 1A). A Student’s T-test showed these differences were statistically significant: wild-type versus SynVL-D, *p* = 0.021; versus SynVL-E/SynVH-C, *p* = 0.011; versus SynVL-E/SynVH-SD, *p* = 0.046). Because the antibody was raised against VL1-44, staining with this antibody in the SynVL-D cohort likely represents staining of the human heavy chain. These results demonstrate that Vλ-expressing OmniChickens produce mature B and T cells that populate the periphery and that presence of human CL did not affect the ability of SynVL-E birds to produce a mature B cell population.

**Table 1:** Student’s T-test, OmniChickens versus wild-type birds.

	Bu1	chIgM	chIgL	huIgL	huVL/VH	TCR1	TCR2/3
**SynVL-D/SynVH-C**	0.67	0.17	0.21	N/A	**0.021**	0.72	1.0
**SynVL-E/SynVH-C**	0.52	0.78	N/A	**0.008**	**0.011**	0.31	0.64
**SynVL-E/SynVH-SD**	0.93	0.88	N/A	**0.035**	**0.046**	0.94	0.57

### Plasma IgY titer is comparable to wild-type birds

**Fig 2:**
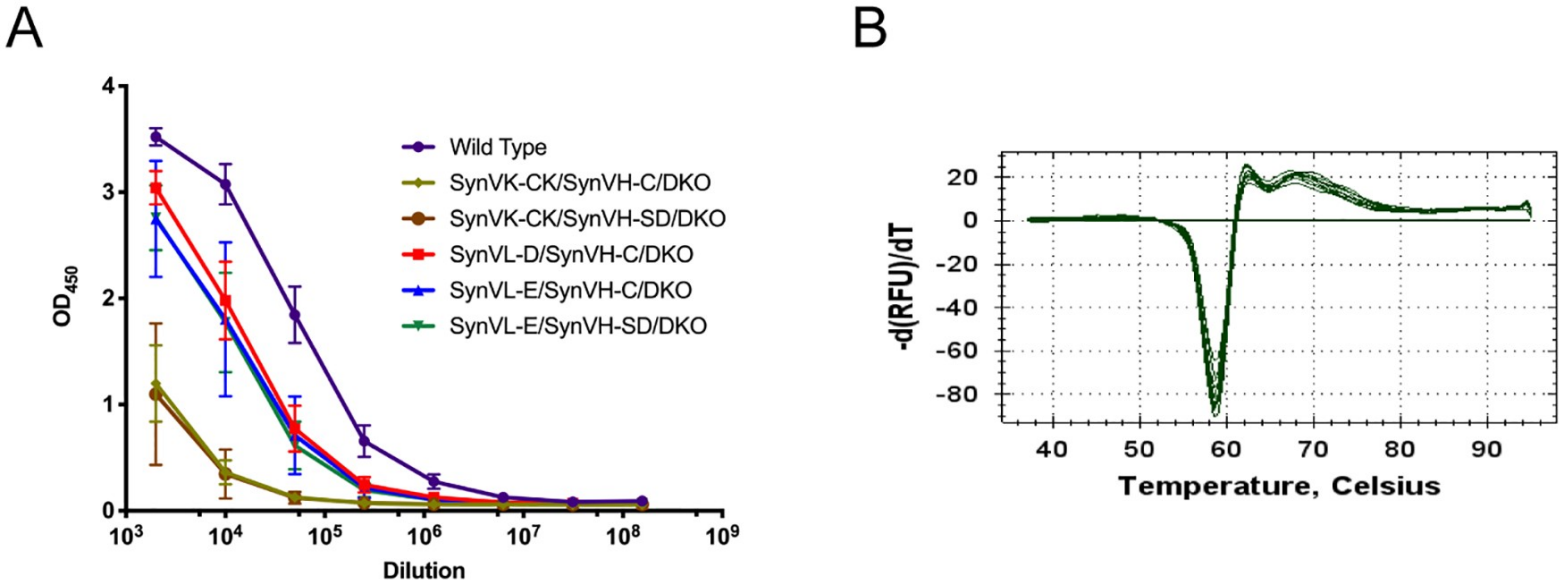
Evaluation of plasma IgY in Vλ OmniChickens compared to wild-type and Vκ OmniChickens. (A) Plasma from 5 week old wild-type (n = 5), SynVK-CK/SynVH-C/DKO (n = 4), SynVK-CK/SynVH-SD/DKO (n = 4), SynVL-D/SynVH-C/DKO (n = 4) and SynVL-E/SynVH-C/DKO (n = 7) and SynVL-E/SynVH-SD/DKO (n = 8) was assessed by non-quantitative IgY sandwich ELISA. Plasma was first diluted 1:1000 in buffer, followed by 5-fold serial dilutions. The results shown are representative of three independent experiments. The standard deviation between birds within a given genotype is shown. (B) In a thermal shift assay recombinantly expressed wild-type and transgenic immunoglobulins show no difference in melting temperature. The graph shown is representative of three independent experiments.

A major question this study aimed to answer was whether or not expression of human Vλ, which more closely resembles the native chicken light chain, would improve the total IgY titer compared to our Vκ (SynVK-CK) OmniChickens. Because SynVL-E has a human CL, we were unable to quantitatively compare IgY titers between this genotype, SynVL-D and SynVK-CK (which both have chCL) and wild-type. Rather, we employed an IgY sandwich ELISA in which both the capture and detecting antibodies were polyclonal. Plasma from SynVK-CK (n = 8), SynVL-D (n = 4), SynVL-E (n = 15) and wild-type birds (n = 5) was collected at 5 weeks and diluted serially (Fig 2A). All three evaluated Vλ genotypes were quite similar, diluting out on average to 10^6^. The wild-type cohort appeared to have a slightly higher total IgY titer, diluting out to approximately 5x10^6^. In contrast, the cohort of Vκ birds diluted out closer to 10^5^. While the Vλ OmniChickens still had a slightly lower IgY titer on average compared to wild-type, the Vκ birds were nearly 10-fold lower. These results suggest that there is a difference between human lambda and kappa light chain expression in our system, but that fusion of chicken CL with human V does not fully explain this difference.

### Thermal stability of IgY

We investigated whether the improved levels of Vλ-containing IgY in OmniChickens as compared to Vκ could be explained at least in part by a possible improvement in protein stability in the chimeric human V/chicken CL (in SynVL-D) or fully human light chain (in SynVL-E). We used a melting curve assay to assess the stability of circulating immunoglobulin from the OmniChicken. Originally developed to measure protein stability under different solvent conditions, the assay employs a highly sensitive fluorescent dye and a real-time PCR unit [20,21]. Briefly, purified protein is incubated in the presence of SYPRO orange and gradually heated in 0.5 °C increments in a BioRad CFX1000. As the protein unfolds, the dye binds the now exposed hydrophobic regions of the protein and fluoresces. The melting temperature of the protein is calculated as the first derivative of relative fluorescence units versus temperature. The assay requires highly purified monomeric protein but aggregation of circulating immunoglobulin following purification from the plasma of different OmniChicken genotypes prevented the analysis. As an alternative, we transfected each transgene combination and wild-type cVH/cVL in an Expi293 serum-free expression system and purified using an IgY affinity column. Compared to wild-type, SynVL-E/SynVH-C and SynVK-CK/SynVH-C had the exact same melting temperature of 58.5 °C (Fig 2B). SynVL-D/SynVH-C had a slightly higher melting temperature of 58.75 °C, suggesting that expressing a human VL on chicken CL does not affect the stability of the mature protein. The difference in total circulating immunoglobulin between kappa and lambda OmniChickens can thus not be explained from a stability standpoint in this particular assay.

### Immune response to a model antigen

With each transgene introduced in our OmniChickens, we have assessed immunocompetence by hyperimmunization of a cohort of animals with a model antigen [9,19]. In this way, we are able to evaluate the immune repertoire and compare different transgenes based on responses to the same protein. For these experiments, we employed human progranulin, a multi-domain protein consisting of 7.5 tandem repeats of the granulin motif, a conserved pattern containing 12 cysteine residues [22]. The precursor protein can be cleaved into its constituent granulins (Grn), A, B, C, D, E, F, G and P, each of which we are able to identify with domain-specific antibodies generated in our wild-type chickens and OmniChickens [13]. Five SynVL-E (three with pre-rearranged and two with the rearranging VH3-23) and three SynVL-D (all with pre-rearranged VH3-23) OmniChickens were immunized with purified, recombinant human progranulin and boosted on a bi-weekly basis. Antigen-specific titer was measured on the off week by ELISA. All animals demonstrated a robust titer at the first blood draw, as shown by representative birds from each genotype (S4 Fig, blue lines). The specific titer continued to increase throughout the immunization, for example, up to 1:1.5 e6 for some birds (S4 Fig, bird 34032). There was no significant difference in response between the SynVL-E OmniChickens expressing SynVH-C versus SynVH-SD. There was also no apparent difference between SynVL-D responses and SynVL-E, suggesting that using a more “chicken-like” VL does not confer any significant advantage during an immune response. The initial immune response and the rapid and steady increase in titer observed with biweekly boosting was also comparable to that observed in our Vκ expressing OmniChickens.

**Fig 3:**
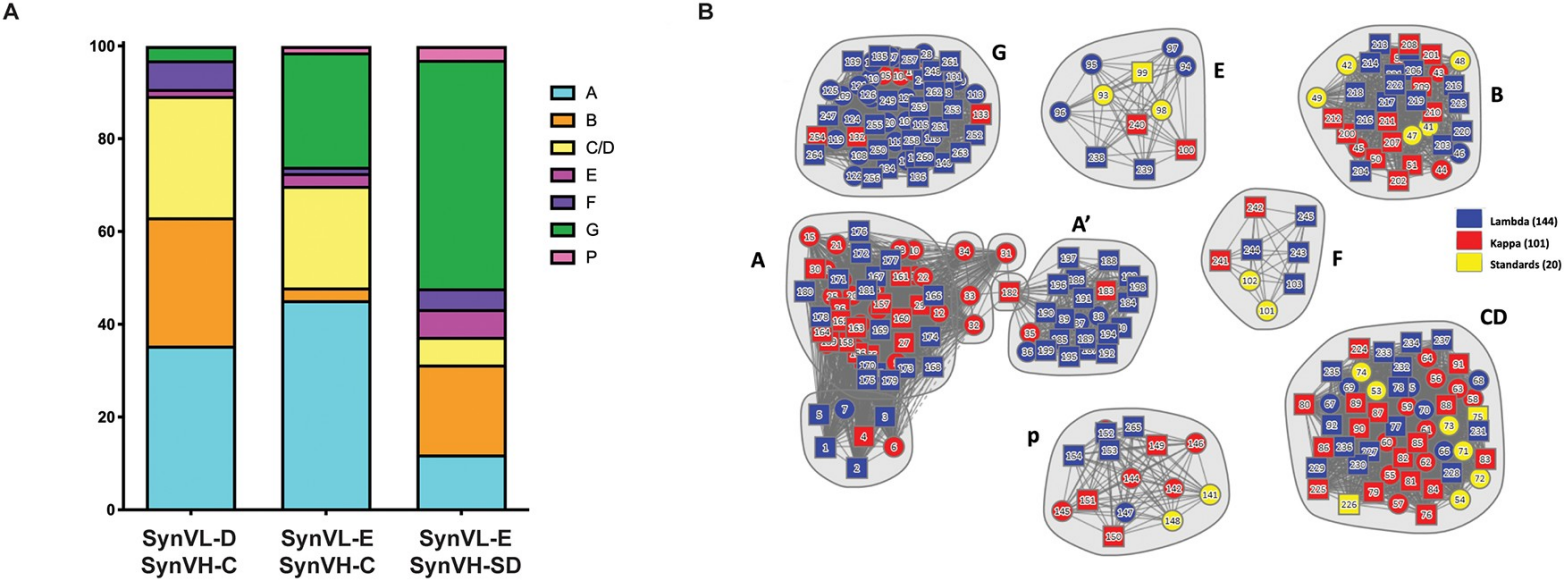
Vλ OmniChickens show broad epitope coverage of progranulin (PGRN), a multi domain model antigen. A cohort of Vλ OmniChickens was immunized with PGRN and antigen specific clones identified using the GEM assay. (A) Epitope binning using high-throughput array SPR demonstrates that SynVL-D recognizes six of 7.5 distinct domains (bar 1) while SynVL-E OmniChickens generated antibodies to all 7.5 domains (bars 2 and 3). (B) A network plot illustrates the relationships between antibodies within a given epitope bin. A cohort of previously described antibodies from Vκ OmniChickens (red) and a set of 20 well characterized “standards” used to identify each Grn domain (yellow) were also included in the array and are shown in the nodal plot. Use of these additional antibodies enabled the identification of a subdomain within GrnA (A’) and also demonstrates the broad epitope space covered by the different OmniChicken genotypes. Vλ-derived antibodies are shown in blue.

All eight immunized OmniChickens were screened using the GEM technology and streptavidin beads coated with biotinylated human progranulin [8,9]. Approximately 200 unique antibodies were recovered from both SynVL-D and SynVL-E. Using high-throughput array SPR, we analyzed the complete cohort in a single experiment. In these experiments, one antibody is immobilized on a chip coated with anti-human Fc and the other antibody is run over the chip as “analyte” either in a premix format with progranulin or after progranulin has been incubated with the immobilized antibodies. Previously, using a chimeric mapping strategy, we identified a set of antibody “standards” to serve as markers for each domain of the progranulin protein [13]. Using these standards, clones to each sub-domain of progranulin were identified from both cohorts of SynVL-E OmniChickens (Fig 3). In the SynVL-D cohort, antibodies were found to all domains except GrnP. GrnA-specific clones dominated (35%) the cohort. The proportion of clones to each subdomain was slightly different between the different SynVL-E genotypes. GrnA, GrnG and GrnC/D-specific clones dominated the SynVL-E/SynVH-C genotype (43%, 24% and 22%, respectively), while GrnG-specific clones dominated the SynVL-E/SynVH-SD genotype (49%) (Fig 3A). We have frequently observed that certain subdomains appear to be less immunogenic than others. GrnF-specific clones are particularly rare. In this study, four GrnF-specific clones were found in each of the SynVL-E cohort and SynVL-D cohorts.

Because we can analyze hundreds of relationships within the antibody cohort using high-throughput SPR, we can not only identify the domain a specific antibody binds, but we can infer subdomains, that is, different epitope specificities within a given domain based on cross blocking relationships. Fig 3B represents these relationships in a nodal plot, where each distinct cluster consists of a grouping of antibodies which all bind the same PGRN domain. Solid lines represent a blocking relationship between two antibodies, while dotted lines represent an asymmetrical block, meaning the blocking relationship was only observed in one direction. This can occur for several reasons including irreversible denaturation of the immobilized antibody during the first regeneration step or poor expression of either the immobilized antibody or the antibody being run as analyte. The majority of blocking relationships, however, could be observed in both directions. In addition to the antibody standards, a subset of the cohort of antibodies described in the original Vκ OmniChicken paper were also included in these experiments. Interestingly, a group of antibodies from the Vλ OmiChickens formed a distinct grouping within the GrnA bin, which we refer to as GrnA’ subdomain. While the GrnA epitope bin consisted of a mix of human specific and mouse cross-reactive clones, the GrnA’ antibodies were all human specific. These results illustrate the diverse repertoire of the OmniChicken. Smaller subdomains were not observed in any of the other primary Grn domains, but further probing of the spleens of these chickens would likely yield increased epitope diversity within each domain.

**Fig 4:**
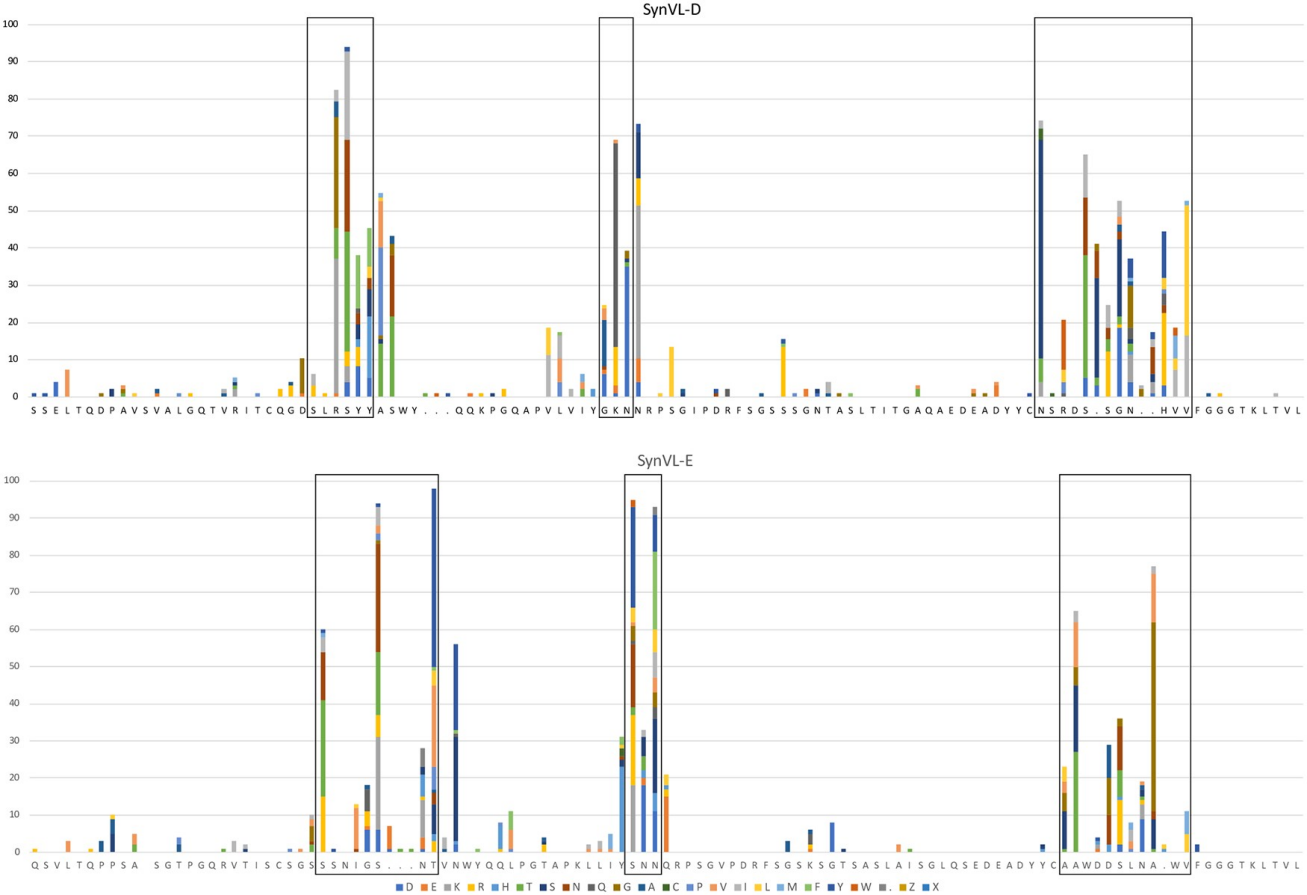
SynVL-D and SynVL-E concentrate amino acid diversity in the CDRs. Sequence diversity is plotted from one hundred clones from both SynVL-D/SynVH-C (top panel) and SynVL-E/SynVH-C (lower panel). The germline sequence for each transgene is shown on the x-axis and amino acid changes at each position are shown as a percentage of the total number of sequences. CDRs (IMGT) are boxed.

Sequence analysis of the light chain showed that the majority of mutations observed were concentrated in the CDRs with minimal changes in the framework regions for both SynVL-E and SynVL-D (Fig 4). Because SynVL-D did not confer any apparent advantage over SynVL-E, we continued our analysis using only clones from the SynVL-E cohort. Gene conversion events are difficult to identify because multiple rounds of gene conversion, with additional changes from random somatic hypermutation, produce quite divergent sequences from the pseudogene array. Some pseudogene sequences could be identified in a number of clones, particularly in CDR-L1 where there are longer stretches of sequence that differ from the germline transgene (S2 Fig).

**Fig 5:**
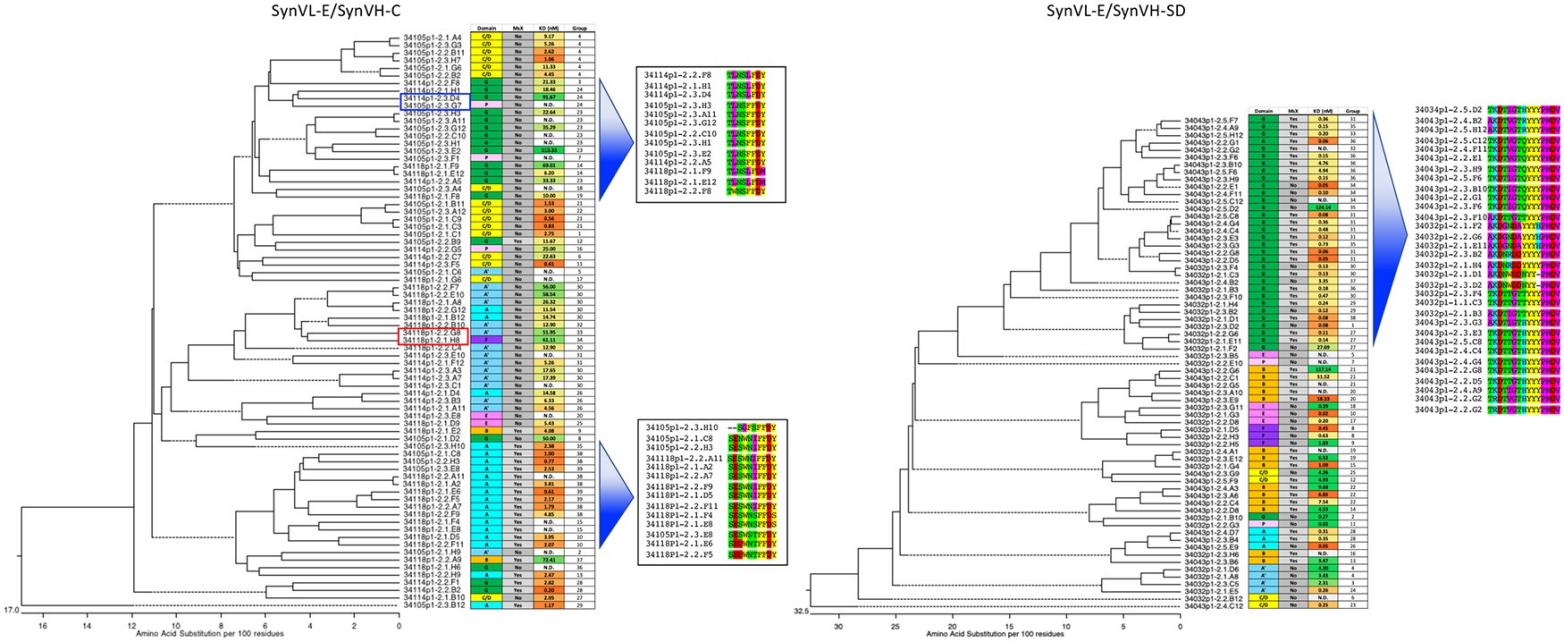
Related clones generally converge on the same epitope bin. A sequence dendrogram based on Vλ-VH is shown beside epitope binning, mouse cross reactivity results and clonotype assignment for SynVL-E/SynVH-C (left) and SynVL-E/SynVH-SD (right). For the SynVL-E/SynVH-C cohort, CDR-H3 is shown for two groupings. A set of GrnG binders (top inset) and a set of GrnA binders (bottom inset) show highly related CDR-H3s. In general, related sequences tend to converge on the same epitope bin, but examples of closely related sequences recognizing different bins could also be seen (red and blue boxes).

We examined the relationship between amino acid sequence and epitope specificity by generating a sequence dendrogram based on both heavy and light chain Vs for each clone (Fig 5). For this analysis, we define clonotype by 100% identity in CDR-H3, assigning each clonotype a number (far right column on each dendrogram). In many cases highly related clonotypes occupy the same epitope bins. In the SynVH-C cohort, for example, the 13 grouped GrnG clones (top of the tree) belong to four highly similar clonotypes, differing only by a few amino acids and suggesting that they derived from a common ancestor (Fig 5, top inset). Despite their similar CDR-H3s, they ranged in affinity from 6.2 nM to over 100 nM against human PGRN. It should be noted that other GrnG-specific clones with unrelated CDR-H3s could also be found in the cohort. The grouping of 14 GrnA binders (bottom of the tree) show a similar pattern as they belong to six related clonotypes (Fig 5, bottom inset). Within this group CDR-H3s differ by only a few amino acid changes. A similar pattern was observed in the SynVH-SD cohort of clones Fig 5, right). A subset of 32 GrnG-specific clones derived from 13 highly related clonotypes.

While we fully expected and observed that highly related sequences tended to cluster around the same epitope bins, there were several examples of clones with different specificities that were still closely related at the protein level. In one extreme example, two antibodies, 34114p1-2.3.D4 and 34105p1-2.3.G7, were the same clonotype, but fell into two different epitope bins (blue box). In most cases, however, closely related sequences that had different epitope specificities did not belong to the same clonotype. For example, clone 34118p1-2.2.G8 and 34118p1-2.1.H8 were 94% identical in their heavy chains, differing in CDR-H3 by one amino acid, but 34118p1-2.2.G8 recognized GrnA’, while 34118p1-2.1.H8 fell into the GrnF epitope bin (Fig 5, red box). Their divergence from each other came primarily from their light chain sequences, which shared only 89% sequence identity.

Although we immunized with and screened on only human progranulin, a significant number of the resulting clones also cross reacted with mouse progranulin, which shares 75% identity with the human homolog. Within the SynVH-C cohort, 28% of clones were mouse cross-reactive, while 58% cross-reacted in the SynVH-SD cohort (Fig 5).

**Fig 6:**
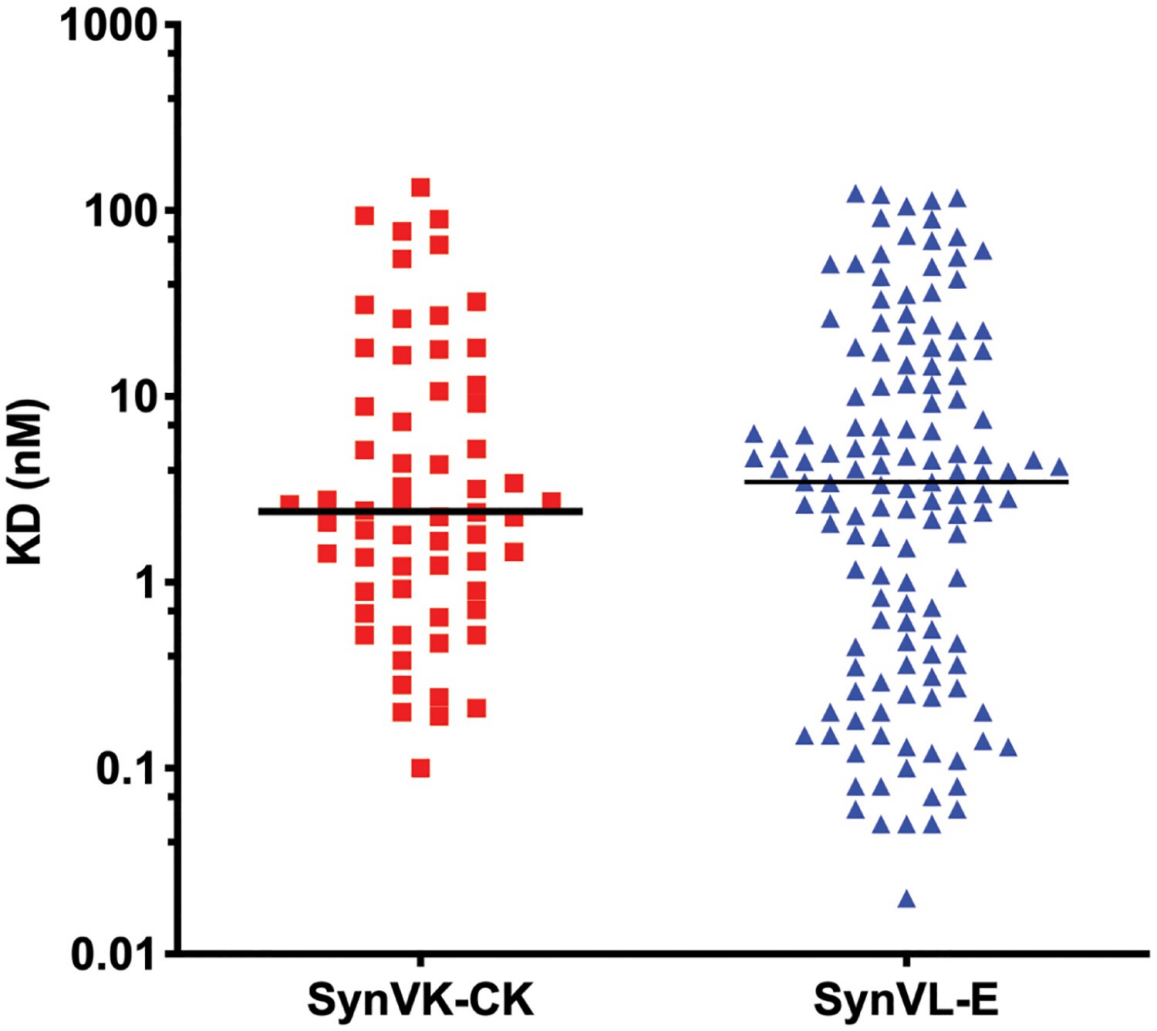
Vλ-derived antibodies reach subnanomolar affinities. Progranulin affinity was measured from a subset of Vλ (right) and previously described Vκ (left) antibodies. The Vκ cohort had a slightly higher median affinity (2.4 nM) compared to the Vλ cohort (3.5 nM), but the difference was not statistically significant (Student’s T-test, *p* = 0.65).

Kinetic analysis was also performed. The median K_D_ was 2.2 nM for human progranulin, and 17.2 nM for mouse progranulin (S5 Fig). Thirty-three percent of the clones displayed subnanomolar affinities for human progranulin, while only a few clones recognizing mouse progranulin displayed an affinity of less than 1 nM. The difference in affinity is not surprising given that the animals were only immunized with human progranulin. A subset of Vλ clones was also run side-by-side with the cohort of Vκ clones from the original study of the Vκ OmniChicken [9]. The median affinity within the Vκ subset was 2.4 nM, while the median affinity for the Vλ subset was 3.5 nM (Fig 6). The difference in affinities between the two cohorts was not statistically significant (Student’s T-test, *p* = 0.65), although the Vλ subset contained a higher proportion of double-digit picomolar clones.

## Discussion

With over 70 approved therapeutic antibodies on the market, the field of monoclonal antibody research continues to expand and evolve [15]. Tracking with this development is the evolution of a number of discovery platforms built on transgenic animals carrying human V regions or novel platforms to rapidly humanize antibodies from alternative species such as sharks or camels [23–26]. The most highly available transgenic platforms are in mice, which have a limited response to proteins with a high degree of sequence conservation between mammalian species. The difficulty of generating cross-reactive antibodies when proteins are highly conserved often necessitates the development of surrogate antibodies. For example, durvulumab, an anti-PD-L1 antibody which is used in the treatment of non-small cell lung cancer, is not mouse cross-reactive [27,28]. This necessitated the development of a surrogate, mouse cross-reactive antibody that could be used in early *in vivo* studies. While the antibody, 10F.9G2 was within 3-fold of the affinity of durvulumab, it was 10-fold less potent as measured by IC_50_. The ability to generate species cross-reactive antibodies at the start of a discovery campaign using phylogenetically distant species like the chicken would eliminate the need to develop such surrogates which cannot perfectly mimic the behavior of the antibody of interest.

The chicken, which is more phylogenetically distant to humans compared to mice and rats, demonstrates robust immune responses to conserved human proteins and frequently generates species cross-reactive antibodies [6,29]. The lack of widespread use of chicken antibodies in clinical research derives from the lack of a fusion partner for chicken B cells, the frequency of disulfide bridges in CDR-H3 [12] and the presence of additional N-glycosylation sites [30]. As a result, chicken monoclonal antibodies have traditionally been derived from phage and other display libraries [31–33]. Subsequent humanization is then required to minimize immunogenicity. We have developed a system to precisely engineer the chicken genome at its heavy and light chain loci in order that human sequence antibodies are produced [9,34–36]. Transgenes were designed to minimize the potential for sequence liabilities in the antibodies. By employing a microdroplet technology we can perform single cell RT-PCR and retain the native VH/VL pairings [8]. We are thus able to exploit the phylogenetic distance of chickens to generate robust antibody responses to human proteins and fully interrogate an *in vivo* affinity matured human sequence repertoire.

To expand the repertoire of the OmniChicken we have generated a new line of birds carrying human Vλ. The targeted insertion of Vλ1–44 or Vλ3–19 at the chicken light chain locus in SynVL-E and SynVL-D birds, respectively, resulted in OmniChickens with normal B and T cell populations in the periphery compared to wild-type chickens as assessed by cell surface staining. Expression of human VL and VH was specifically detected using a polyclonal antibody raised against our selected germline V regions. Thus, as seen in our Vκ-expressing OmniChickens, the insertion of human Vλ does not appear to affect the development and migration of B and T cells into the periphery. Consistent with these observations, the Vλ OmniChickens performed well when challenged with our model antigen, human progranulin. Like wild-type birds immunized with progranulin, the Vλ animals displayed a robust immune response which was maintained throughout the immunization protocol. This was not surprising; we have looked at over 30 different antigens and typically observe an early, robust immune response.

Interrogation of the SynVL-E cohort of antibodies using high-throughput SPR demonstrated complete epitope coverage of the human progranulin protein while the SynVL-D cohort produced antibodies directed at seven out of eight domains. The elusive domain for SynVL-D, GrnP, is half the size of the other domains, and consistent with that, appears to be less immunogenic than the other domains. In our numerous studies using progranulin as a model antigen, we typically observe only a small number of clones specific for GrnP [9]. While it appeared that different epitope bins were immunodominant for each genotype, it would be premature to conclude that these patterns represented differences in immune recognition encoded by the transgenes. The sample size from each genotype was small (<100 antibodies), and certain clonotypes appeared to dominate the larger populated bins, giving the appearance of epitope bias. In each of the birds we used for this project, we screened less than 5% of the total splenocyte population of each bird. Additional rounds of screening with the GEM assay would most likely introduce more clonal diversity into the antibody cohort.

Significant interest in the use of a divergent species for therapeutic antibody discovery stems from the difficulty in generating species cross-reactive antibodies in mice. As an alternative, surrogate antibodies, raised in phylogenetically distant animals, that closely mimic the binding characteristics of the clinical candidate must be identified [37,38]. Not only does this often involve a second antibody discovery campaign but may also require the generation of a knockout to break tolerance [39]. Recently, Gjetting at al. described an anti-PD1 antibody derived from wild-type chickens [40]. The group first identified over 100 unique antibodies and generated up to four humanized variants for each clone, finally identifying their lead candidate, Sym021. Because Sym021 bound human, mouse and cynomolgus monkey PD1 with high-affinity (30, 810 and 110 pM, respectively), surrogate antibody development was unnecessary. In addition, Sym021 bound human PD1 with affinity several orders of magnitude higher than clinically approved pembrolizumab and nivolumab (2800 and 800 pM, respectively). Their results demonstrate the powerful ability of the chicken to generate high-affinity antibodies to unique epitopes in comparison to mice, and also the relative ease with which species cross-reactive antibodies are generated in the chicken. The OmniChicken retains these advantages but bypasses the need for humanization and the generation of multiple humanized variants as it directly delivers human sequence antibodies.

The single framework design of the functional V and corresponding pseudogenes in the OmniChicken minimizes diversity in the framework regions and reduces the potential number of sequence liabilities. In particular, the proportion of cysteine residues in CDR-H3 is higher in wild-type chickens than humans. Wu et al. reported the fraction of cysteines out of the total amino acid content of CDR-H3 at 9.4% in wild-type chickens versus 1.6% in humans [12]. Because the V regions of the OmniChicken are entirely human, we rarely observe non-canonical cysteine residues [19]. In fact, deep sequencing of the antibody repertoire of a cohort of OmniChickens showed less than 1.5% cysteine content in CDR-H3. While significant technological advances have been made to interrogate wild-type chicken repertoires, the OmniChicken bypasses many of the technical challenges, such as the prevalence of cysteines in the CDRs.

In contrast to the Vκ-expressing OmniChicken, which has a lower total IgY titer than wild-type birds, Vλ OmniChickens showed a total IgY titer comparable to wild-type by ELISA. Because the Vλ OmniChickens contain human sequences in the Vλ region, or in both the Vλ and CL regions, and thus lack light chain epitopes that would be recognized by the polyclonal anti-IgY (H+L) detection antibodies, we were unable to directly compare the Vλ titers to wild-type titers. A semi-quantitative ELISA, however, showed that the Vλ birds showed a titer more comparable to wild-type than our Vκ birds. As there was no difference in titer between the fully human light chain, SynVL-E, and the chimeric light chain, SynVL-D (hVL-chCL), the expression of human light chain V regions with chicken CL does not appear to be detrimental to IgY expression. It is unlikely that the chimeric heavy chain expressed in the OmniChicken is the culprit of the lower IgY titer in the Vκ birds, as it is the same heavy chain expressed in both κ and λ birds. In thermodynamic studies using recombinantly expressed protein, all of our expressed transgenes showed a similar melting temperature to wild-type IgY. While we may have missed subtle differences in melting temperature due to the sensitivity of our assay, there is evidence that, in the context of a Fab, the primary role of CH1/Cλ is not the overall stabilization of the molecule [41]. In their study, Toughiri et al. expressed a scFv (VH/Vλ) derived from PGT128, a broadly neutralizing antibody that recognizes the glycan shield of HIV and a series of chimeric Fabs, exchanging CH1 and Cλ for CH1/Cκ or Cα/Cβ of TCRα/β. The PGT128 Fab with native CH1/ Cλ had a small increase in thermal stability (T_m_ +6°C) compared to the scFv, but the substitution of CH1/Cκ or Cα/Cβ had basically the same effect. In addition, expression of the Fv with any of these constant domain combinations significantly decreased protein aggregation, suggesting that the primary role of CH1/Cλ might be to shield hydrophobic residues at the interface. Taken together, these findings suggest that the expression of chicken CH1 and chicken CL may not significantly influence the overall stability of the molecule and thus may not explain the difference in total IgY between our Vλ and Vκ OmniChickens. With our current reagents, we cannot fully explain the difference in titer between our Vκ and Vλ expressing OmniChickens. We are investigating other possible explanations for this discrepancy in titer and are currently pursuing crystallization and structural studies. It is formally possible that the difference may stem from the transcriptional or translational level. This is less likely, however, as the only region of the locus we have replaced is the V-region. The untranslated regions are all chicken.

In conclusion, the addition of the lambda light chain to the existing OmniChicken platform increases the overall diversity of antibody repertoires that can be mined for therapeutic leads. The fact that human lambda is more closely related to chicken IgL may allow it to function more efficiently than a chimeric human kappa (hVK/cCL) within the context of the chicken immune system. However, once the affinity-matured V genes are cloned into a fully human recombinant antibody format, we find that both OmniChicken light chain isotypes are equally able to generate high quality antibody candidates.

## Materials and methods

### Constructs

Production of the IgL and IgH knockouts was previously described [35,36]. The human lambda constructs, SynVL-D and SynVL-E consist of functional V regions containing pre-rearranged human VL3-19/JL2 and VL1-44/JL3, respectively. The pre-rearranged functional V regions were designed by joining the germline human V and J genes, in-frame, with no nucleotide additions or deletions. These “germline” V regions can be found in the normal human repertoire. For the VL pseudogenes, a diverse set of CDRs was selected from the naturally occurring somatic human sequences found in the NCBI EST database, which was queried with the VL3-19 and VL1-44 germline gene. VL3-based ESTs were used as the source for the SynVL-D pseudogenes, and VL1-based ESTs were used for the SynVL-E pseudogenes. Individual pseudogenes may consist of CDRs from different ESTs, and the frameworks were all identical to the matched functional V region in each construct. Framework 4 sequence was not included in the pseudogenes. The constant regions were chicken CL for SynVL-D and human CL3 for SynVL-E. The human heavy chain constructs have been described elsewhere [9,19,36].

### Germ cell culture

Germ cell derivation and culture were performed as previously described [34,42,43]. Re-derived knock out cell lines IgL KO 229–92 and IgH KO 472–138 were transfected with the SynVL and SynVH constructs, respectively, using phiC31 integrase as described [44].

### Breeding

Animal experiments described in this manuscript were done in an Association for Assessment and Accreditation of Laboratory Animal Care (AAALAC) accredited facility in accordance with Ligand Pharmaceuticals’ Institutional Animal Care and Use Committee (IACUC) approved protocols and under supervision of the IACUC committee. To obtain OmniChicken birds with the genotype SynVλ/IgL KO; SynVH/IgH KO, crosses between SynVλ/+; IgH KO/+ and IgL KO/+; SynVH/+ birds were performed. Progeny were genotyped by PCR using DNA obtained from comb biopsy. Males and females were kept for analysis.

### Flow cytometry

PBMCs were isolated using histopaque-1077 (Sigma, 10771), labeled on ice for 1h with the following antibodies from Southern Biotech: ms anti-Bu1 (8395), ms anti-ch IgM (1020), ms anti-ch IgL(8340), ms anti-ch ΤCR-γδ (8230), ms anti-TCRαβ/Vβ1 (8240) or ms anti-TCRαβ/Vβ2 (8250), diluted in 1%BSA-PBS, washed and then incubated with AF-647 anti-ms IgG (Jackson Immunoresearch, 715-605-150). PBLs were used at a concentration of 1 x 10^6^ cells/mL. Data was collected using an Attune Acoustic Focusing Cytometer (Thermo Fisher) and single lymphocytes (gated on FSH v. FSA) were analyzed using FloJo. The plots shown are representative of several independent experiments. A two-tailed Student’s T-test was used for statistical analysis using GraphPad Prism.

### Assessment of plasma IgY

Blood was drawn from 5-week-old birds into EDTA-containing collection tubes. Samples were centrifuged at 4 °C and plasma collected. ELISA plates were coated with rb anti-ch IgY (Sigma, C2288) at 2 ug/mL in PBS, washed in PBS/0.5% Tween-20 (PBST), and blocked for 1h in PBST/3% nonfat dry milk. The blocking buffer was aspirated and serial dilutions of plasma in blocking buffer added for 1h at RT. Plates were washed, incubated with anti-ch IgY HRP at 0.1 ug/mL (Sigma, A9046) for 1h at room temperature, washed, and developed with TMB (Thermo Fisher, 002023) for 10min, and the reaction stopped with 1N HCl. Plates were read at 450 nm on a BioTek microplate reader.

### Assessment of antigen specific titer

To assess antigen-specific titer, ELISA plates were coated with 2 ug/mL human progranulin (Sino Biologicals, 10826-H08H) in PBS. Plates were blocked as described above, incubated with plasma at the indicated dilutions for 1h, washed, incubated with rb anti-ch IgY-HRP (Sigma, A9046) for 1h washed, and developed as above. During the immunization period, antigen specific titer is measured on a bi-weekly basis. For each assessment, the previous draw and the preimmune sample are also run on the ELISA plate.

### Thermostability assay

The open reading frames of the different VL and VH insertions were cloned into the pF5A mammalian expression vector (Promega, C940A) and co-transfected in the Expi293 Expression System (Thermo Fisher, A14635). Briefly, 1 ug of VL DNA and 1 ug VH DNA were incubated for 25 min at room temperature with 5.4 uL Expifectamine transfection reagent in a total volume of 200 uL of OptiMem (Thermo Fisher, 31985062). Complexed DNA was then added to 2 mL cultures of Expi293 cells seeded at 1e6 cells/mL. Enhancers were added 15 hrs post transfection, and supernatants were harvested at 72 hrs.

Because all heavy chain insertions contain chicken constant regions, supernatants were purified using anti-chicken IgY purification columns (LigaTrap, LT-142) according to the manufacturer’s instructions. Equal volumes of purified supernatants were then added to 25 uL of SYPRO orange protein gel stain (Life Technologies, S6650) diluted 1:400 in PBS. Samples were then ramped from 37 °C to 95 °C in 5 °C increments with a fluorescence reading every 5 sec. Relative fluorescence units (RFU) are graphed versus temperature, and the melting temperature is determined from the plot of the first derivative of RFU versus temperature.

### Immunizations

Recombinant human PGRN was obtained from Sino Biologicals. For the initial immunization, 200 ug of protein was emulsified in 250uL of Freund’s Complete Adjuvant (Thermo Fisher, 77140) and injected into the breast tissue. Fourteen days later, chickens were boosted with 100 ug protein and 250uL of Freund’s Incomplete Adjuvant (Thermo Fisher,77145). Animals were subsequently boosted every other week, with blood drawn on the off-week to assess antigen-specific titer. Four days before euthanization and spleen harvesting, animals received a final IV boost with no adjuvant.

### Single B cell GEM screening

We employed a single lymphocyte screening method, the Gel-Encapsulated Microenvironment (GEM) assay (US Patents 8030095 and 84151738) to identify antigen-specific mAbs from immunized chickens. 5μm aldehyde-latex beads (Thermo Fisher, A37306) were coated with streptavidin followed by biotinylated antigen, overnight, blocked with 3% milk-PBS, and tested by labeling with plasma from immunized animals. GEMs were prepared containing a single secreting B cell and antigen-coated beads and incubated for 3h at 37°C in RPMI/10%FCS containing 2ug/ml AF-594 anti-ch IgY (Thermo Fisher, A11042) as previously described [8].

### Single cell PCR to amplify VH and VK regions and clone as scFvFc

Cells secreting antigen-specific mAbs were captured and their VH and VK regions were amplified by a two-step, semi-nested strategy as described [8]. The variable regions were assembled with human Fc by overlap extension PCR.

### Expression of scFvFc antibodies

Recombinant scFvFc was expressed in Expi293 cells as described [8].

### Biosensor analysis

Binding kinetics and affinities were determined as described previously using a capture kinetic assay format by Array SPR imaging using the CFM (Carterra, USA) and MX96 (Ibis, Netherlands) [9].

Interaction analysis studies were performed by high-throughput surface plasmon resonance (SPR) on Carterra’s LSA platform equipped with HC-30M or HCX-30M (pre-activated) sensor chips at 25°C and in a run buffer of 10 mM Hepes pH 7.4, 150 mM NaCl, 3 mM EDTA, and 0.05% Tween-20 (HBSET) supplemented with 1 g/l BSA. Epitope binning was performed in a “classical sandwich” assay format as described previously [45] with the following modifications. To prepare the surfaces for these experiments, the LSA’s single flow cell (SFC) and 96-channel printhead (96PH) were primed in a run buffer of 25 mM MES pH 5.5 (Carterra) and 0.01% Tween-20. When using the HC-30M chip type, the entire surface was activated in the SFC for 10 mins with a freshly prepared 1:1:1 v/v/v mixture of 0.4 M 1-ethyl-3-(3-dimethylaminopropyl)carbodiimide (EDC, Pierce) + 0.1 M N-hydroxysulfosuccinimide (sulfo-NHS, Pierce) + 0.1 M MES pH 5.5. The antibodies (for use as ligand) were then coupled in batches of 96 via the 96PH and by addressing all 4 print block locations in a serial manner, allowing 5-min contact time per print block, a 384-ligand array was created. Antibodies were used directly from crude supernatant and typically diluted 100-fold (aiming for final antibody concentration of approximately 1 μg/ml) into 10 mM sodium acetate pH 4.5 + 0.01% Tween-20 coupling buffer (Carterra). The 96PH was returned to water for cleaning, and any unreactive esters on the printed chip were quenched with 1 M ethanolamine pH 8.5 (Carterra) for 7 min in the SFC. When using the HCX-30M chip type, no activation step was required. The coupled chip was then primed via the SFC in a run buffer of HBSET and 1 g/l BSA and binning was performed by injecting 30 nM recombinant purified human progranulin with His-tag (R&D systems) as antigen for 3 min, followed immediately by an antibody analyte for 3 min, typically using a 10-fold dilution of the supernatant in the run buffer. After every binning cycle, the arrayed antibodies were regenerated with 75 mM phosphoric acid (2x 30-sec pulses). By merging the antibody cohort with a panel of purified mAb standards from the WT chicken [13], we inferred the subdomain (or “granulin”) specificities of the new cohort by their cross-blockade of the standards. As a complementary approach, the subdomain assignments of the human-specific antibodies were determined using a chimeric swap epitope mapping strategy by monitoring the binding specificity of the antibody analytes over spots coupled with human PGRN, mouse PGRN, and five previously described human/mouse chimeras [13]. Epitope binning and mapping were therefore performed in the same experiment taking advantage of the large ligand capacity of the chip, which allowed for up to 384 unique ligands (antibodies or antigen variants) per chip. Binning and mapping data were analyzed in Carterra’s Epitope software to generate heat maps and network blocking plots.

Binding affinities were determined in a “capture kinetic” assay format using a chip coupled with goat anti-human IgG Fc-specific polyclonal (Southern Biotech) as the capture reagent. To prepare the surfaces for these experiments, the LSA’s SFC and 96PH microfluidic modules were both primed in a run buffer of HBSET. The capture surface was prepared as a “lawn” via the SFC by activating the chip with EDC/sulfo-NHS/MES (as above) for 10 min, coupling the goat anti-human reagent at 50 μg/ml in 10 mM sodium acetate pH 4.3 + 0.01% Tween-20 for 15 min and quenching with 1 M ethanolamine pH 8.5 for 7 min. The coupled chip was then used to capture the antibodies (diluted up to 100-fold in HBSET) in batches of 96 via the 96PH, to create a 384-ligand array, as above. The purified WT mAb standards were diluted to 5 μg/ml and captured in parallel to enable a direct comparison. Having arrayed the antibodies, the SFC was docked onto the chip and primed with a run buffer of HBSET + 1 g/l BSA. The recombinant purified PGRN was prepared as a 3-fold dilution series spanning 0.4–300 nM and samples were injected for 3 min each in ascending concentration, allowing a 20-min dissociation phase. Prior to injecting these analyte samples, several buffer samples were injected to stabilize the newly captured surfaces and provide “blank” analyte cycles for double-referencing (data processing) purposes[45]. Carterra’s kinetic software was used to process and analyze the data globally using a simple Langmuir binding model. The affinity or equilibrium dissociation constant (*K*_D_) for PGRN interacting with each captured mAb (per spot), was determined by the ratio of the kinetic rate constants, *K*_D_ = *k*_d_/*k*_a_.

## Supporting information

S1 Fig SynVL-D and SynVL-E are inserted into the chicken IgL locus. The wild-type chicken IgL locus consists of a single V and J downstream of the VL pseudogene array (top). A knockout of the locus was generated which deleted the chicken V, J and C, and inserted an attP site. An array of pseudogenes based on human ESTs along with pre-rearranged VL3-19 (SynVL-D, middle) or pre-rearranged VL1-44 (SynVL-E, bottom) and the native chicken CL or human CL were inserted at the attP site previously placed in the locus. Selectable markers were removed by Cre recombination, leaving behind a single attP site and an attR site. Human sequences are indicated in red, chicken in blue.
(TIFF)

S2 Fig The SynVL-E pseudogene array represents a diverse subset of ESTs. CDRs from 20 naturally occurring human ESTs (NCBI) with VL1-44 framework were cloned for the SynVL-E pseudogene array. Any framework changes in the ESTs were changed back to the germline sequence. The SynVL-D pseudogene array followed a similar strategy. The inset shows several CDRL1 sequences from antigen specific clones in which specific pseudogenes could be partially identified.
(TIF)

S3 Fig SynVL-E-expressing birds do not stain with anti-ch IgL. SynVL-E birds carry a fully human lambda chain and do not stain with anti-chicken IgL. A representative SynVL-E bird shows staining with the chicken B cell marker Bu1 (left) and with an anti-huVL/VH antibody (right) but no staining with anti-ch IgL (center).
(TIF)

S4 Fig Vλ OmniChickens display robust titers against human progranulin. Representative titers from SynVL-D/SynVH-C (left), SynVL-E/SynVH-C (middle) and SynVL-E/SynVH-SD (right). Titers were taken on a bi-weekly basis and measured by ELISA.
(TIF)

S5 Fig OmniChicken derived antibodies against progranulin are in the single digit nanomolar range. Affinities were measured against human progranulin (175 antibodies; left) and mouse progranulin (79 antibodies, which represents the subset of antibodies that are mouse cross-reactive; right). The median affinities against human and mouse PGRN were 2.2 nM and 17.2 nM, respectively. The results are representative of several independent experiments.
(TIF)
