# Supplementary figures and images for: Expression of human lambda expands the repertoire of OmniChickens
Source: PLoS One. 2020 Jan 29;15(1):e0228164. doi: 10.1371/journal.pone.0228164 (PMC6988971; doi:10.1371/journal.pone.0228164)

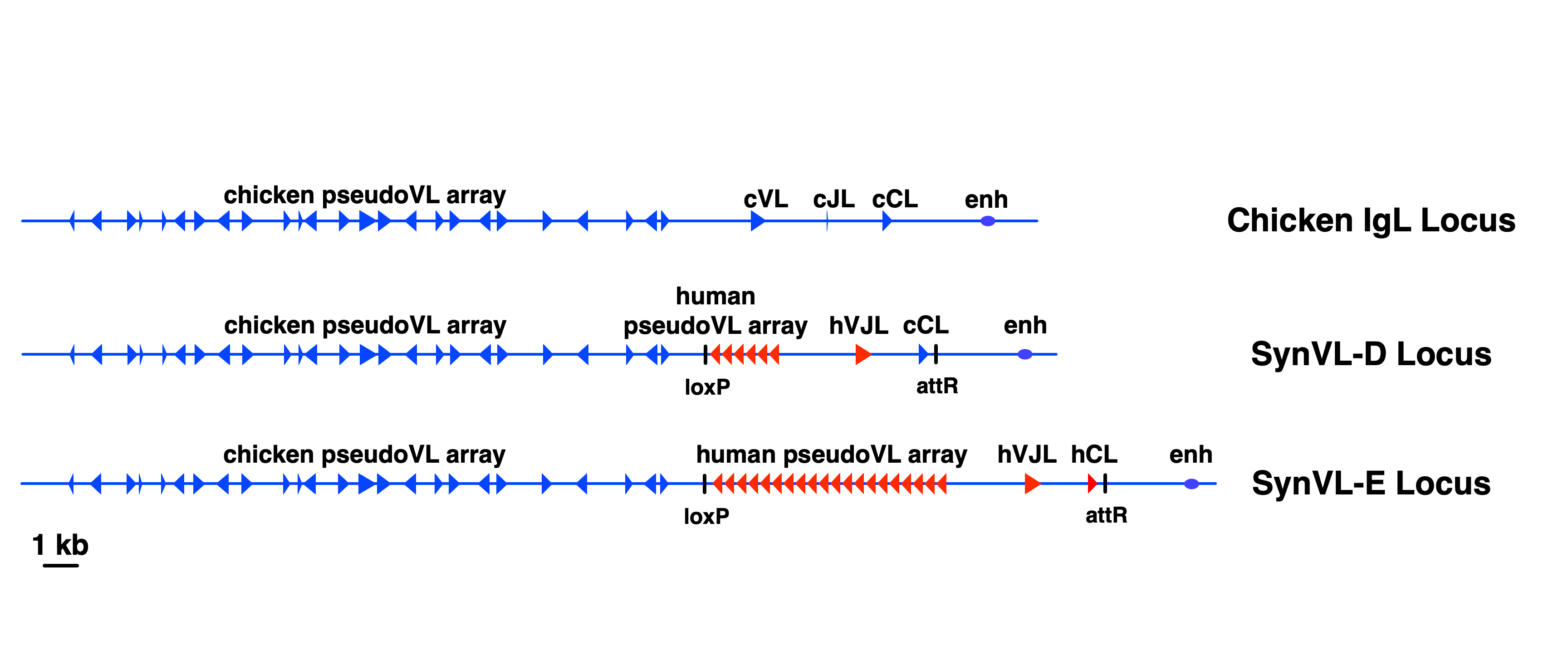

Supplement: S1 Fig — The wild-type chicken IgL locus consists of a single V and J downstream of the VL pseudogene array (top). A knockout of the locus was generated which deleted the chicken V, J and C, and inserted an attP site. An array of pseudogenes based on human ESTs along with pre-rearranged VL3-19 (SynVL-D, middle) or pre-rearranged VL1-44 (SynVL-E, bottom) and the native chicken CL or human CL were inserted at the attP site previously placed in the locus. Selectable markers were removed by Cre recombination, leaving behind a single attP site and an attR site. Human sequences are indicated in red, chicken in blue. (TIFF) [file pone.0228164.s001.tiff]

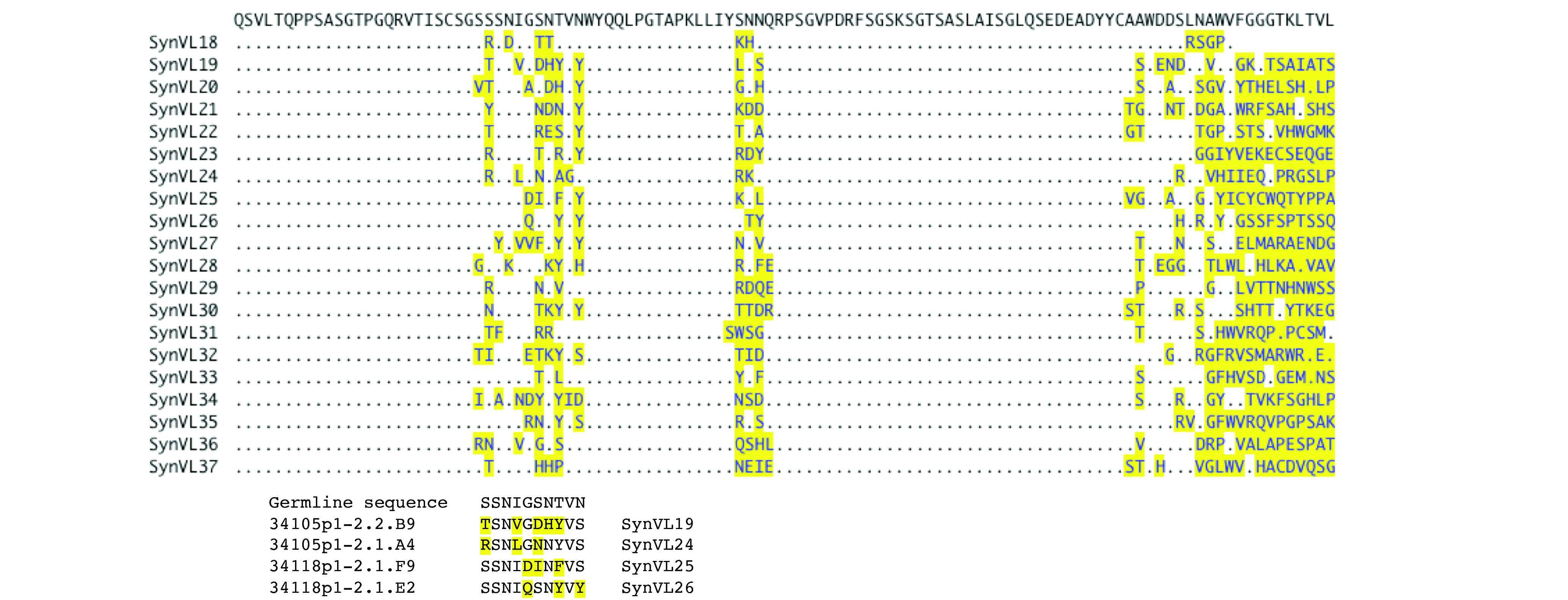

Supplement: S2 Fig — CDRs from 20 naturally occurring human ESTs (NCBI) with VL1-44 framework were cloned for the SynVL-E pseudogene array. Any framework changes in the ESTs were changed back to the germline sequence. The SynVL-D pseudogene array followed a similar strategy. The inset shows several CDRL1 sequences from antigen specific clones in which specific pseudogenes could be partially identified. (TIF) [file pone.0228164.s002.tif]

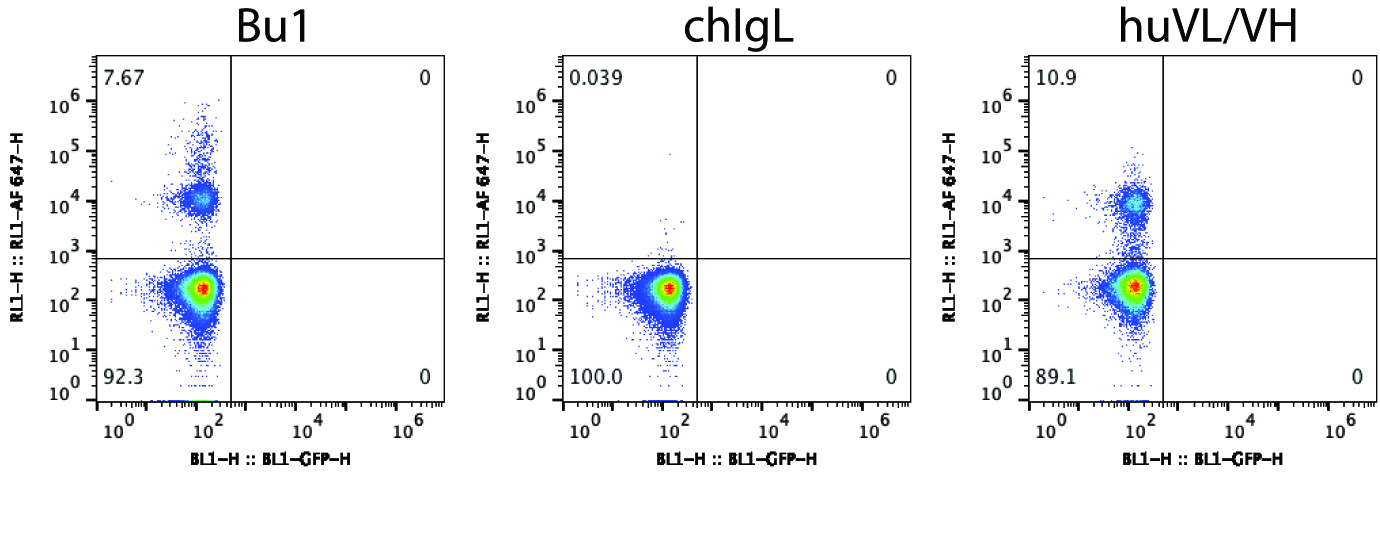

Supplement: S3 Fig — SynVL-E birds carry a fully human lambda chain and do not stain with anti-chicken IgL. A representative SynVL-E bird shows staining with the chicken B cell marker Bu1 (left) and with an anti-huVL/VH antibody (right) but no staining with anti-ch IgL (center). (TIF) [file pone.0228164.s003.tif]

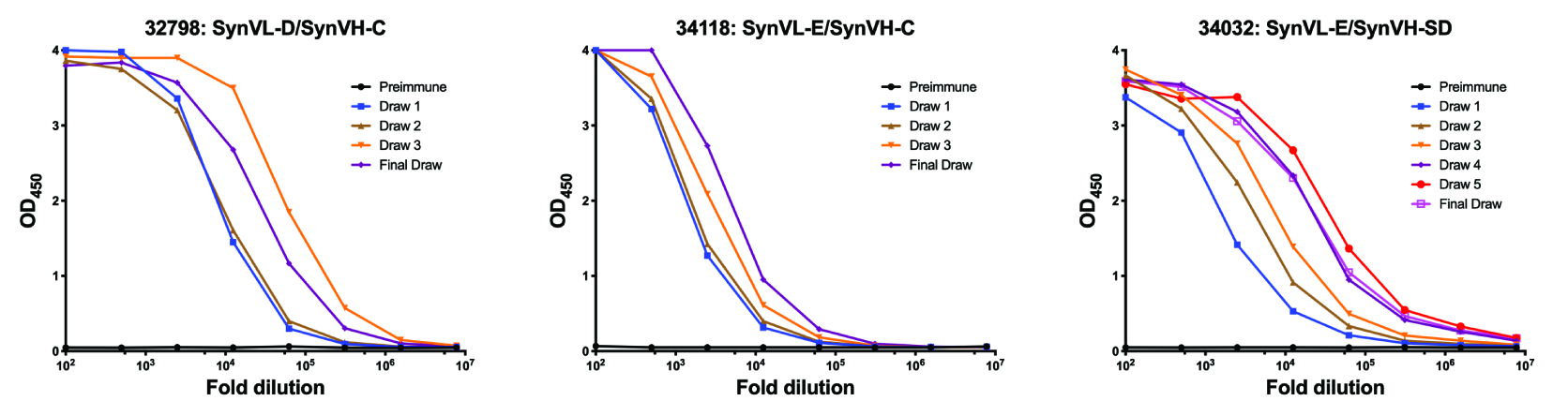

Supplement: S4 Fig — Representative titers from SynVL-D/SynVH-C (left), SynVL-E/SynVH-C (middle) and SynVL-E/SynVH-SD (right). Titers were taken on a bi-weekly basis and measured by ELISA. (TIF) [file pone.0228164.s004.tif]

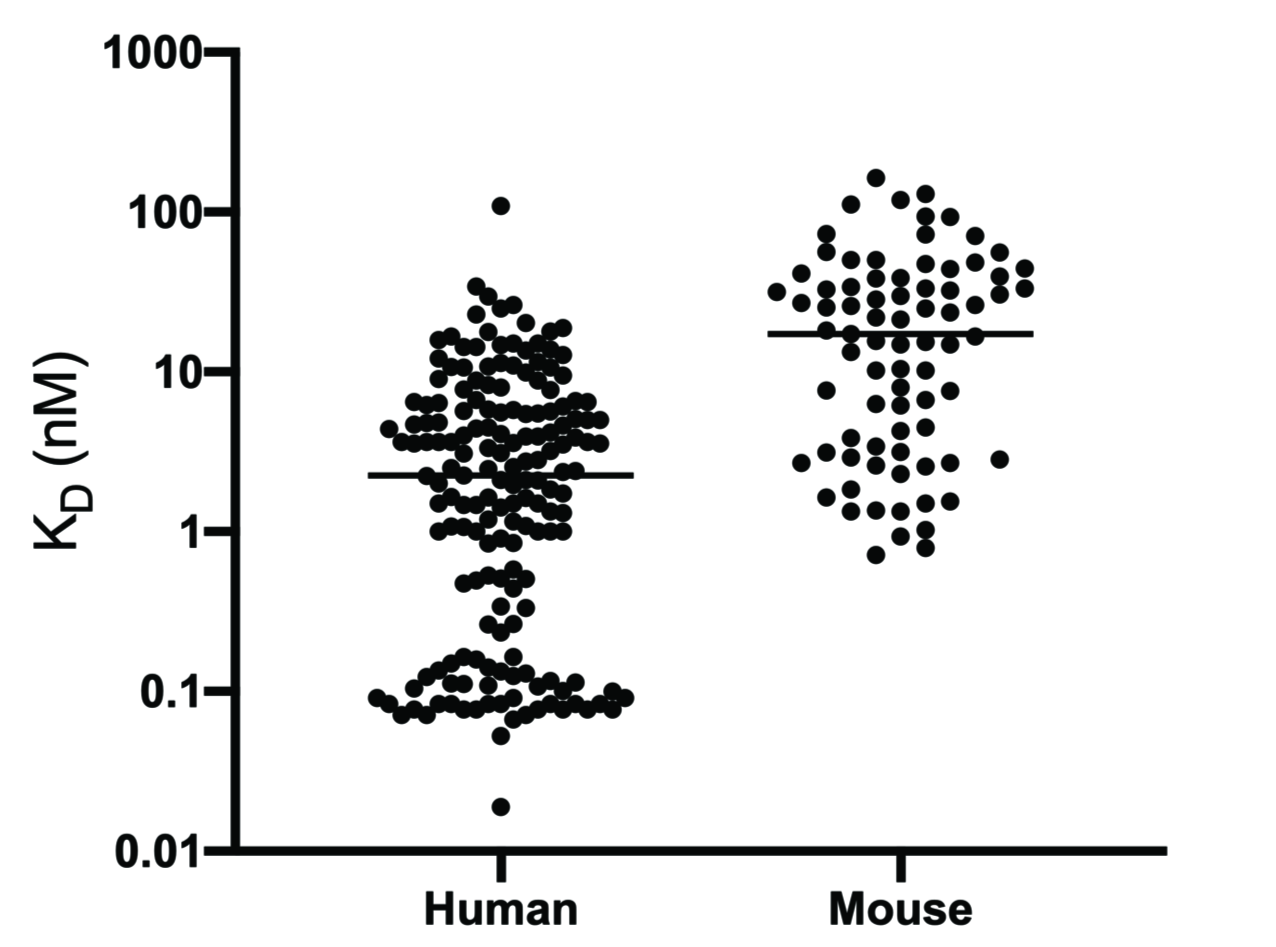

Supplement: S5 Fig — Affinities were measured against human progranulin (175 antibodies; left) and mouse progranulin (79 antibodies, which represents the subset of antibodies that are mouse cross-reactive; right). The median affinities against human and mouse PGRN were 2.2 nM and 17.2 nM, respectively. The results are representative of several independent experiments. (TIF) [file pone.0228164.s005.tif]
